# Cyclic Ether Contaminant Removal from Water Using Nonporous Adaptive Pillararene Crystals via Host-Guest Complexation at the Solid-Solution Interface

**DOI:** 10.34133/2019/5406365

**Published:** 2019-05-12

**Authors:** Yujuan Zhou, Kecheng Jie, Run Zhao, Errui Li, Feihe Huang

**Affiliations:** State Key Laboratory of Chemical Engineering, Center for Chemistry of High-Performance & Novel Materials, Department of Chemistry, Zhejiang University, Hangzhou 310027, China

## Abstract

The removal of soluble cyclic ether contaminants, such as dioxane and THF, produced in industrial chemical processes from water is of great importance for environmental protection and human health. Here we report that nonporous adaptive crystals of perethylated pillar[5]arene (**EtP5**) and pillar[6]arene (**EtP6**) work as adsorbents for cyclic ether contaminant removal via host-guest complexation at the solid-solution interface. Nonporous** EtP6** crystals have the ability to adsorb dioxane from water with the formation of 1:2 host-guest complex crystals, while** EtP5** crystals cannot. However, both guest-free** EtP5** and** EtP6** crystals remove THF from water with** EtP5** having a better capacity. This is because** EtP5** forms a 1:2 host-guest complex with THF via host-guest complexation at the solid-solution interface while** EtP6** forms a 1:1 host-guest complex with THF.** EtP6** also shows the ability to selectively remove dioxane from water even in the presence of THF. Moreover, the reversible transitions between nonporous guest-free** EtP5** and** EtP6** structures and guest-loaded structures make them highly recyclable.

## 1. Introduction

1,4-Dioxane, a cyclic ether often simply called dioxane, is primarily used as a solvent in industry as well as in the laboratory and a stabilizer for the transport of halogenated hydrocarbons [1]. Dioxane is also a by-product of the polyester manufacturing process, leading to its subsequent occurrence in industrial wastewater streams [2, 3]. Nevertheless, dioxane is also known as a highly stable contaminant and potential carcinogen in water and is becoming a threat for human and animal health [4–6]. There has been severe dioxane pollution in history. During 1976-1985, leakage of dioxane occurred in Ann Arbor, Michigan, and severely damaged the drinking water [7–9]. The removal or degradation of dioxane has not been completed until now. Some efforts have been devoted to increasing control, removal, and remediation of dioxane from sources of pollution. Recent methods involve electrolysis and ozonation [2, 10], phytoremediation [11], advanced oxidation processes (AOPS) [12–15], and so on. However, it is still challenging to completely remove dioxane due to its high miscibility with water, low vapor pressure, and nonbiodegradable nature. Moreover, these current methods are complex, highly energy-consuming, and unrecyclable. Thus, the search for new and easy strategies or adsorbents for adsorption and subsequent removal of dioxane from water is of great importance.

Pillar[*n*]arenes are a new and important class of macrocyclic hosts [16, 17]. They are highly symmetrical and rigid, easy to chemically modify, and possess abundant host-guest properties [18–26]. Recently, our group pioneered research on nonporous adaptive crystals (NACs) of pillararenes [27–32]. These nonporous crystals with “intrinsic porosity” can capture specific vaporized guests that have noncovalent interactions with them to form new guest-loaded crystal structures, that is, host-guest chemistry at the solid-gas interface. Based on these unique properties, NACs of pillararenes have been successfully applied in the adsorptive separations of hydrocarbons such as styrene purification and xylene isomer separation [28, 30]. However, the host-guest chemistry of NACs at the solid-solution interface still remains unexplored. The development of such properties for pillararene NACs may broaden their applications in more areas such as liquid-phase separation and water treatment.

**Figure 1:**
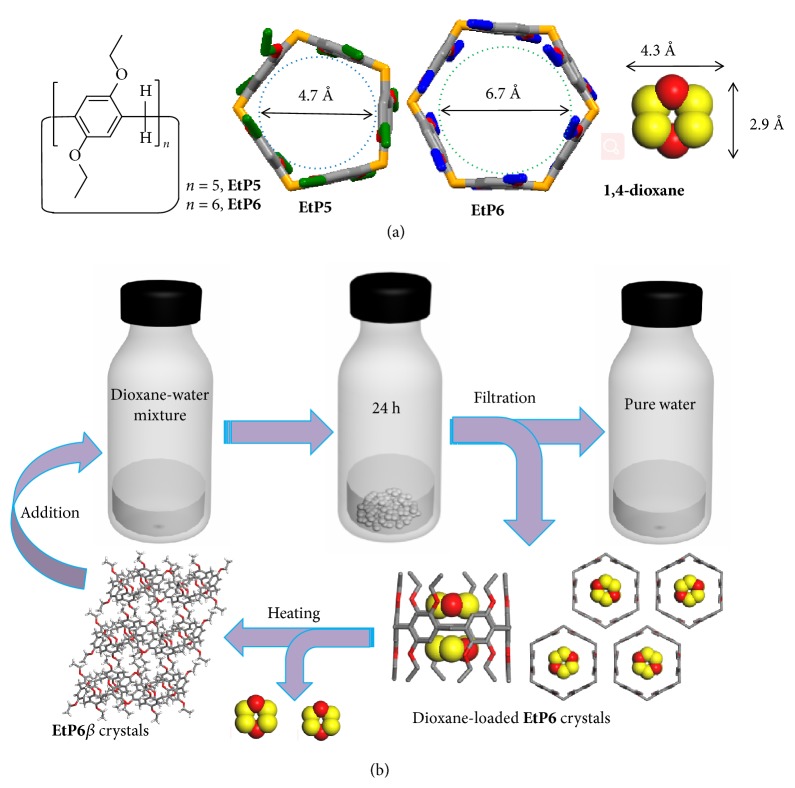
*Chemicals used here and schematic representation of* **EtP6** *adsorption.* (a) Chemical structures and cartoon representations of** EtP5**,** EtP6,** and 1,4-dioxane. (b) Schematic representation of** EtP6** as an absorbent for dioxane capture and the recycling of** EtP6**.

Herein, we found that NACs of pillararenes worked as adsorbents to remove cyclic ether contaminants, such as dioxane and THF, from water* via* host-guest complexation at the solid-solution interface. Two easily obtained pillararenes, perethylated pillar[5]arene (**EtP5**) and pillar[6]arene (**EtP6**), were selected and used as adsorbents. Guest-free** EtP6** crystals were found to have the ability to adsorb dioxane from water while** EtP5** crystals cannot. Adsorption of dioxane from water led to a structural transition of** EtP6** from a guest-free EtP6 structure (**EtP6***β*) to a dioxane-loaded 1:2 host-guest complex (2(dioxane)@**EtP6**, Figure 1). However, both guest-free** EtP5** and** EtP6** crystals removed THF from water via solid-solution host-guest complexation with** EtP5** crystals having a better capacity. That is because guest-free** EtP5** crystals (**EtP5***α*) form a 1:2 host-guest complex with THF (2(THF)@**EtP5**) at the solid-solution interface while** EtP6***β* crystals only form 1:1 host-guest complex with THF (THF@**EtP6**).** EtP6** also shows the ability to selectively remove dioxane from water even in the presence of THF. Upon removal of guests from the host-guest complex crystals, both** EtP5** and** EtP6** are transformed back to their original guest-free states and can be recycled many times without degradation.

## 2. Results 

### 2.1. Preparation of Guest-Free Pillararenes

**EtP5** and** EtP6** (Figure 1) were synthesized according to previous reports [18, 27–31]. To use** EtP5** and** EtP6** as adsorbents, guest-free samples of** EtP5** and** EtP6** were obtained (the detailed method is given in the supplementary file). Powder X-ray diffraction (PXRD) experiments showed that both activated** EtP5** and** EtP6** were crystalline in the solid state (referred to as** EtP5***α* and** EtP6***β*, respectively). Synchrotron X-ray diffraction experiments were performed to illustrate their single crystal structures. Both** EtP5***α* and** EtP6***β* show rearrangements of the pillar structures and the loss of their cavities (Figures [Supplementary-material supplementary-material-1]–[Supplementary-material supplementary-material-1]) [30]. Meanwhile, the densely packed arrangement of pillararene units leads to nonporosity of** EtP5***α* and** EtP6***β* as confirmed by N_2_ sorption experiments (Figures [Supplementary-material supplementary-material-1]-[Supplementary-material supplementary-material-1]).

### 2.2. Dioxane Removal Experiments

**Figure 2:**
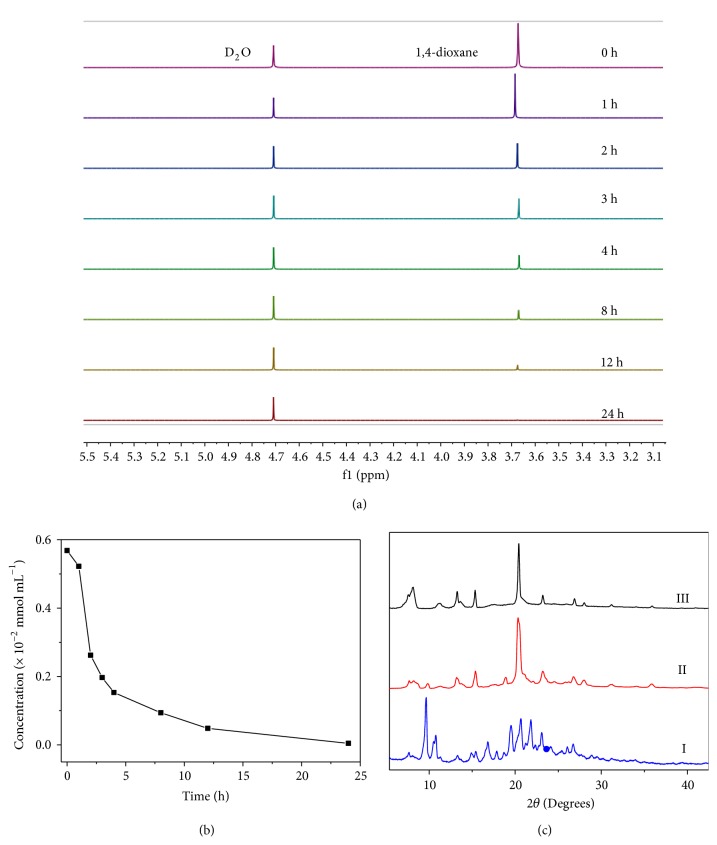
*Investigations on* **EtP6** *adsorption of dioxane from water.* (a) Time-dependent partial ^1^H NMR spectra (400 MHz, D_2_O, 25°C) of a 0.500 mg mL^−1^ dioxane-D_2_O solution upon addition of** EtP6***β*. (b) Time-dependent dioxane concentration change in D_2_O upon addition of** EtP6***β*. (c) PXRD patterns: (I)** EtP6***β*; (II)** EtP6***β* after filtration from the 0.500 mg mL^−1^ dioxane-D_2_O solution; (III)** EtP6***β* after filtration from a 1.00 mg mL^−1^ dioxane-D_2_O solution.

Despite their nonporosity, we investigated the dioxane adsorption abilities of** EtP5***α* and** EtP6***β* from water, respectively. To do so, dioxane was dissolved in D_2_O (0.600 mL) with a concentration of 0.500 mg mL^−1^ (5.7 × 10^−3^ mmol mL^−1^), and 1.00 mg of water-insoluble** EtP5***α* and** EtP6***β* crystals were added, respectively. As can be seen from the time-dependent ^1^H NMR spectra, the peak related to dioxane barely changed after addition of** EtP5***α* (Figures [Supplementary-material supplementary-material-1]-[Supplementary-material supplementary-material-1]). However, after addition of** EtP6***β*, the peak of dioxane decreased over time and almost completely disappeared after 24 hours (Figure 2(a)). The final concentration of dioxane after adsorption was calculated to be 4.37 × 10^−5^ mmol mL^−1^, about 130 times lower than the original concentration (Figures 2(b), [Supplementary-material supplementary-material-1]). The adsorption efficiency of dioxane reached 99.2%, indicating the highly efficient adsorption capacity of** EtP6***β* (Figures 2(b), [Supplementary-material supplementary-material-1]). Upon addition of another 5.00 mg of** EtP6***β* into the solution, the final concentration of dioxane was calculated to be 4.37 × 10^−6^ mmol mL^−1^ (0.413 mg L^−1^) after 24 hours ([Supplementary-material supplementary-material-1]), which is under the discharge limit for 1,4-dioxane of the Korean Ministry of Environment (5.00 mg L^−1^) [8]. This phenomenon indicated that** EtP6***β* instead of** EtP5***α* can remove dioxane from water effectively.

To understand the adsorption mechanism, both** EtP5***α* and** EtP6***β* were filtered from the dioxane aqueous solutions 24 hours after they were immersed. ^1^H NMR spectra in CDCl_3_ showed that no new peaks appeared for** EtP5***α*, while a dioxane peak appeared for** EtP6***β* (Figures [Supplementary-material supplementary-material-1], [Supplementary-material supplementary-material-1]). The amount of dioxane can be calculated as two dioxane molecules per** EtP6** molecule (mole/**EtP6**). Moreover, compared with the ^1^H NMR spectrum of dioxane in CDCl_3_, the dioxane peak has no chemical shift change in the presence of** EtP6** ([Supplementary-material supplementary-material-1]). This implies that** EtP6** does not have host-guest interactions with dioxane in solution due to the presence of CDCl_3_ molecules as competitive guests. However, the weak host-guest interactions between** EtP6** and dioxane may emerge at the solid-liquid interface because of the absence of competitive guests, thus facilitating** EtP6***β* crystals to capture dioxane from water. Thermogravimetric (TG) analyses also confirmed the results. There is no apparent weight loss below 400°C for** EtP5***α* after immersion in the dioxane solution, indicating that no dioxane was adsorbed in** EtP5***α* ([Supplementary-material supplementary-material-1]). However, an apparent weight loss (13.1%) below 160°C for** EtP6***β* occurred after being soaked in the dioxane-water solution, which can also be calculated to be two moles/**EtP6** ([Supplementary-material supplementary-material-1]). These results are thus in accordance with NMR. Powder X-ray diffraction (PXRD) experiments were then performed to monitor the structural information. For** EtP5***α*, the PXRD pattern did not change after immersion in the dioxane solution, meaning no structural transitions ([Supplementary-material supplementary-material-1]). For** EtP6***β*, the PXRD pattern after immersion in the dioxane-water solution was different from the original one, but with a reservation of several small original peaks (Figure 2(c), II). Moreover, the PXRD pattern was completely changed after immersion in a higher dioxane-water solution with a concentration of 1.00 mg mL^−1^ (Figure 2(c), III). These results indicated the occurrence of structural transitions from guest-free** EtP6***β* to a dioxane-loaded new structure after adsorption of dioxane from water.

**Figure 3:**
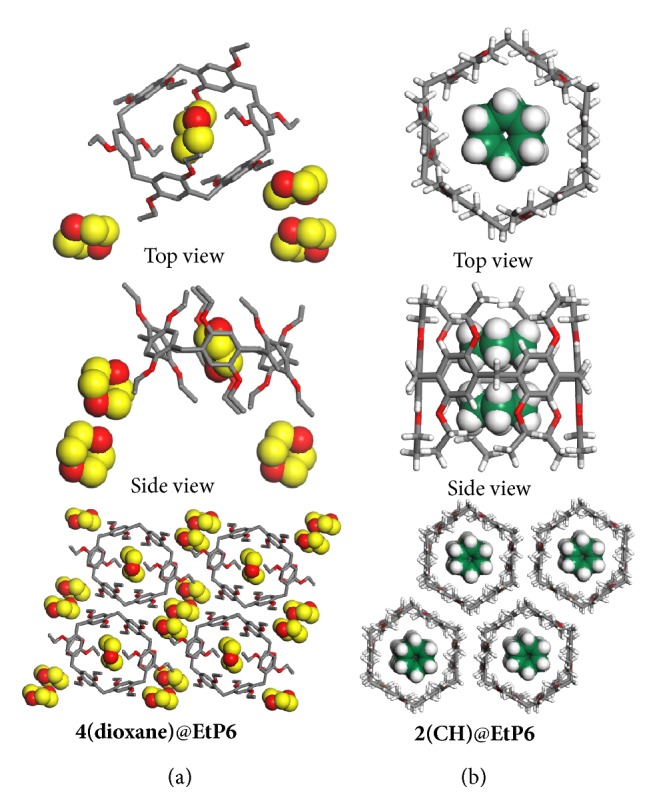
*Crystal structures of host-guest complexes.* Single crystal structures: (a) 4(dioxane)@**EtP6**; (b) 2(**CH**)@**EtP6** [25]. Here** CH** represents cyclohexane.

To reveal the new structure of** EtP6**, dioxane-loaded** EtP6** single crystals were obtained by a solution-growth method and characterized by X-ray crystallography. To our surprise, in the crystal structure of solution-grown dioxane-loaded** EtP6** (4(dioxane)@**EtP6**, Figure 3(a)), four dioxane molecules correspond to one** EtP6** molecule with one located in the cavity and three outside the cavity. Meanwhile, the hexagonal shape of** EtP6** is deformed to some extent. The deformed hexagonal pillar structure of** EtP6** assembles a window-to-window packing mode, leading to the formation of infinite intrinsic 1D channels with dioxane inside and outside the channels (Figure 3(b), right). It should be worth noting that the ratio of dioxane to** EtP6** in the single crystal structure is twice of that obtained from** EtP6***β* capturing dioxane from water. Moreover, the PXRD pattern of** EtP6***β* after capturing dioxane from water is totally different from the one simulated from the single crystal structure of 4(dioxane)@**EtP6** ([Supplementary-material supplementary-material-1]). These results implied that after capturing dioxane from water,** EtP6***β* was transformed into a new structure that is unlike the solution-grown dioxane-loaded** EtP6** structure. We then focused on a previously reported cyclohexane (**CH**)-loaded** EtP6** crystal structure (2(**CH**)@**EtP6**) with two** CH** molecules per** EtP6** molecule [25], the same ratio as** EtP6***β* after capturing dioxane from water. The PXRD pattern of** EtP6***β* after capturing dioxane matched well with that simulated from 2(**CH**)@**EtP6**, manifesting their structural similarities ([Supplementary-material supplementary-material-1]). Hence, we conclude that after capturing dioxane,** EtP6***β* was transformed into a honeycomb-like structure with two dioxane molecules located in the cavity of one** EtP6** molecule.

### 2.3. Tetrahydrofuran Removal Experiments

**Figure 4:**
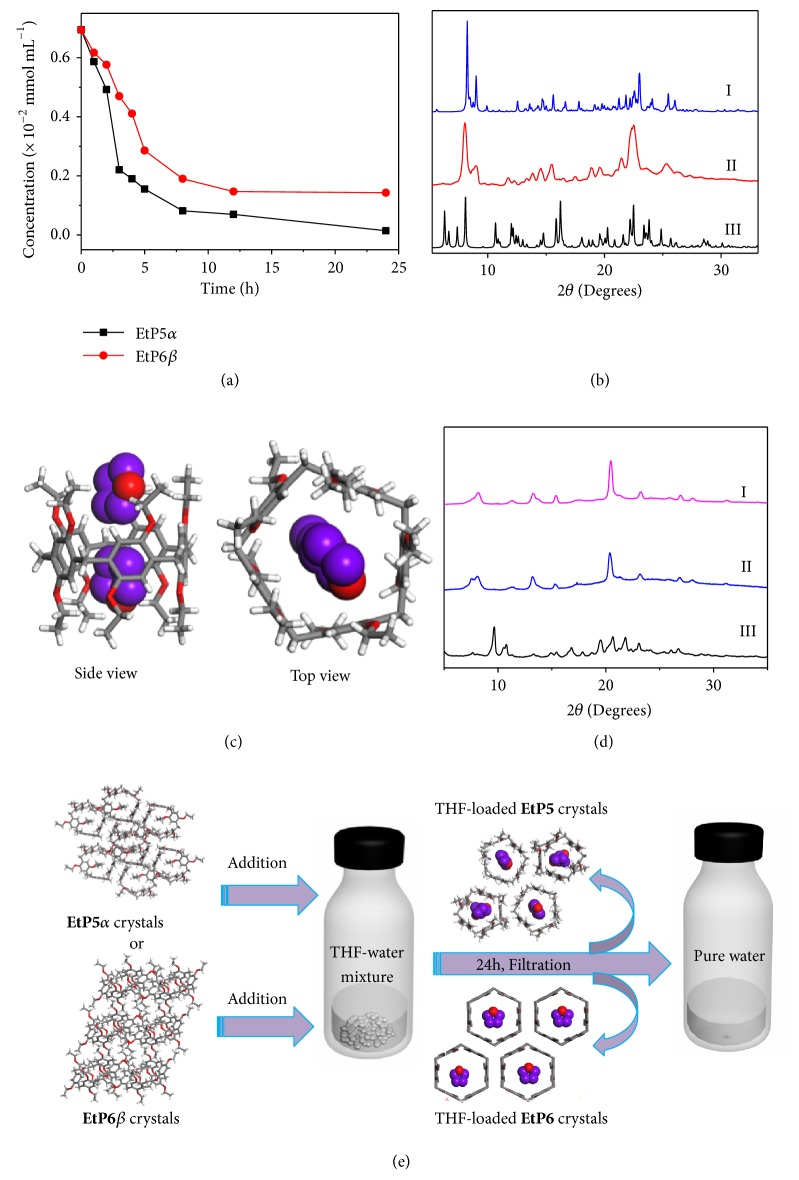
*Investigations on* **EtP5** *and* **EtP6** *adsorption of THF from water.* (a) Time-dependent THF concentration change in D_2_O upon addition of** EtP5***α* and** EtP6***β*. (b) PXRD patterns: (I) simulated from single crystal structure of 2(THF)@**EtP5** [28]; (II)** EtP5***α* after filtration from a 0.500 mg mL^−1^ THF-D_2_O solution; (III)** EtP5***α*. (c) Single crystal structures: 2(THF)@**EtP5**. (d) PXRD patterns: (I)** EtP6***β* after filtration from a 1.00 mg mL^−1^ dioxane-D_2_O solution; (II)** EtP6***β* after filtration from a 0.500 mg mL^−1^ THF-D_2_O solution; (III)** EtP6***β*. (e) Schematic representation of** EtP5***α* and** EtP6***β* as absorbents to remove THF from water.

Tetrahydrofuran (THF), another cyclic ether pollutant with a smaller molecular size, is also encountered in many chemical processes [33]. THF can react readily with oxygen to produce an unstable hydroperoxide. Distillation of peroxide containing THF increases the peroxide concentration, resulting in a serious risk of explosion. THF also forms an azeotrope with water and the mixture of THF-water needs separation during the manufacture of THF [34, 35]. Although** EtP5** cannot remove dioxane from water presumably due to size effect of host-guest complexation at the solid-liquid interface, its potential in the removal of THF was explored. Upon addition of** EtP5***α* crystals (1.00 mg) to 0.600 mL of D_2_O with a THF concentration of 0.500 mg mL^−1^ (6.90 × 10^-3 ^mmol mL^−1^), the time-dependent ^1^H NMR spectra showed that the peaks of THF decreased over time and almost completely disappeared after 24 hours (Figures [Supplementary-material supplementary-material-1]-[Supplementary-material supplementary-material-1]). The final concentration of THF after adsorption was calculated to be 1.42 × 10^−4^ mmol mL^−1^, about 49 times lower than the original concentration (Figure 4(a)). Interestingly,** EtP6***β* also showed the ability to remove THF from water (Supplementary [Supplementary-material supplementary-material-1]). However, the final concentration of THF after treatment with** EtP6***β* was 1.43 × 10^−3^ mmol mL^−1^, much higher than that with** EtP5***α* (Figure 4(a)). Upon addition of another 1.00 mg of** EtP5***α* or** EtP6***β* into the respective solutions, the final concentrations of THF after 24 hours were calculated to be 7.15 × 10^−6^ mmol mL^−1^ and 1.02 × 10^−4^ mmol mL^−1^ (Figures [Supplementary-material supplementary-material-1], [Supplementary-material supplementary-material-1]), respectively. These results indicate that although both** EtP5***α* and** EtP6***β* can remove THF from water, the efficiency of** EtP5***α* (98.0%) was much higher than that of** EtP6***β* (79.5%).

After filtration from the THF-water solution, both** EtP5** and** EtP6** crystals were characterized by ^1^H NMR, TGA, and PXRD. ^1^H NMR of both crystals dissolved in CDCl_3_ showed clear peaks related to THF (Figures [Supplementary-material supplementary-material-1], [Supplementary-material supplementary-material-1]). The molar ratios of THF to** EtP5** and** EtP6** were calculated to be 2:1 and 1:1, respectively. This suggests the reason why** EtP5** had a better performance in the THF removal. Similar to the case in the dioxane removal, the THF peaks in the presence of either** EtP5** or** EtP6** have no chemical shift changes compared with those of single THF in CDCl_3_, indicating the absence of host-guest interactions of THF with either** EtP5** or** EtP6** in solution (Figures [Supplementary-material supplementary-material-1]–[Supplementary-material supplementary-material-1]). Thus, the weak host-guest interactions that happen at the solid-liquid interface without competitive guests may be the driving force for** EtP5***α* and** EtP6***β* crystals to capture THF in water. TG analyses also showed similar results to that obtained by NMR. The weight loss below 120°C can also be calculated as 2 and 1 THF molecules per host molecule, respectively (Figures [Supplementary-material supplementary-material-1]–[Supplementary-material supplementary-material-1]). PXRD experiments showed that both** EtP5***α* and** EtP6***β* underwent structural changes after immersion in the THF-water solution. The PXRD pattern of** EtP5***α* was completely changed to a new one and matched the pattern simulated from the single crystal structure of THF-loaded** EtP5** (2(THF)@**EtP5**, Figure 4(c)) [28], indicating the structural transition from** EtP5***α* to 2(THF)@**EtP5** after adsorption of THF from water. Interestingly, the PXRD pattern of** EtP6***β* after adsorption of THF became similar to that of** EtP6***β* after immersion in the dioxane-water solution (Figure 4(d)), manifesting their structural similarities. Thus, it can be deduced that the THF-loaded** EtP6** (THF@**EtP6**) is also a honeycomb-like structure but with 1:1 rather than 1:2 host-guest complex.

### 2.4. Selective Removal of Dioxane in the Presence of THF

**Figure 5:**
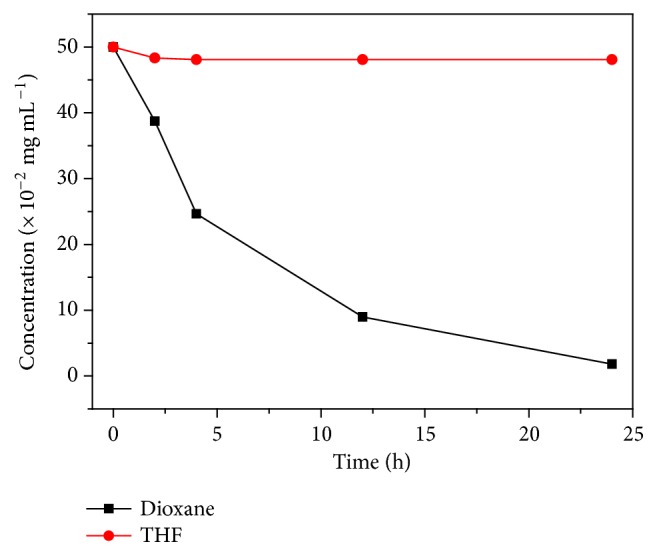
*Selective removal of dioxane in the presence of THF.* Time-dependent dioxane and THF concentration changes in D_2_O upon addition of** EtP6***β*.

Since** EtP6***β* can remove dioxane and THF individually from water, we wondered whether it could selectively remove THF or dioxane from an aqueous solution containing both THF and dioxane. Upon addition of** EtP6***β* (5.00 mg) to a THF/dioxane/D_2_O mixture (both the weight concentrations of THF and dioxane were 0.500 mg mL^−1^; the total volume of the mixture was 0.600 mL), the time-dependent ^1^H NMR spectra ([Supplementary-material supplementary-material-1]) showed that the concentration of dioxane decreased over time while THF almost remained the same. After 24 hours, the final concentration of dioxane was calculated to be 0.018 mg mL^−1^ while the concentration of THF remained as high as 0.480 mg mL^−1^ (Figure 5). These results implied that** EtP6***β* can remove dioxane from water even in the presence of THF with high selectivity.

### 2.5. Recyclability

**Figure 6:**
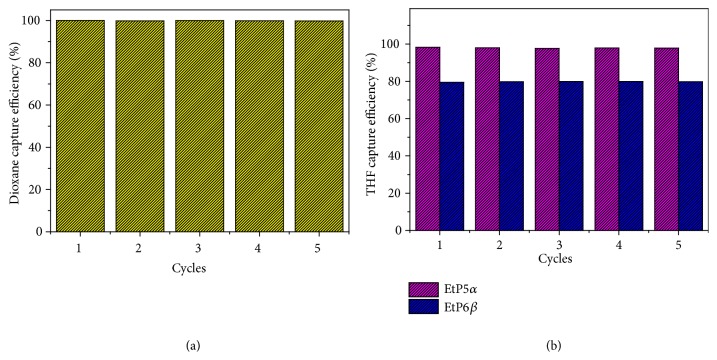
*Recyclability of* **EtP5** *and* **EtP6** *crystals.* (a) Dioxane capture efficiency after** EtP6** is recycled five times. (b) THF capture efficiency after** EtP5** or** EtP6** is recycled five times.

One shortcoming of common adsorbents is the decreased performance over time due to fouling. In practical use, an adsorbent must be recycled without any degradation. Upon heating to completely remove dioxane guests from 2(dioxane)@**EtP6**, the PXRD pattern showed that the desolvated 2(dioxane)@**EtP6** was transformed back to** EtP6***β* ([Supplementary-material supplementary-material-1], II and III). Similar phenomena were also observed for 2(THF)@**EtP5** and THF@**EtP6**. PXRD experiments confirmed the complete removal of THF from 2(THF)@**EtP5** and THF@**EtP6** (Figures [Supplementary-material supplementary-material-1], [Supplementary-material supplementary-material-1]), respectively. Furthermore, the recovered** EtP5***α* and** EtP6***β* remove THF and dioxane from water again, respectively, without degradation after recycling five times (Figure 6). Thus, we can conclude that reversible host-guest complexation at the solid-liquid interface contributes to the recyclability of pillararene crystals.

## 3. Discussion

In summary, we found that nonporous adaptive pillararene crystals,** EtP5***α* and** EtP6***β*, can be used as adsorbents to remove cyclic ethers from water via host-guest complexation at the solid-solution interface.** EtP6***β* crystals have the ability to adsorb dioxane from water while** EtP5***α* crystals cannot. Adsorption of dioxane leads to a structural transition of** EtP6** from** EtP6***β* to a 1:2 host-guest complex 2(dioxane)@**EtP6**. However, both** EtP5***α* and** EtP6***β* crystals remove THF from water via host-guest complexation at the solid-solution interface with** EtP5***α* having a better capacity. This is due to the formation of a 1:2 host-guest complex of** EtP5** with THF (2(THF)@**EtP5**) rather than the 1:1 host-guest complex of** EtP6** with THF (THF@**EtP6**).** EtP6***β* also shows the ability to selectively remove dioxane from water even in the presence of THF. Compared with current methods to remove dioxane and THF, this approach via host-guest recognition at the solid-liquid interface has several advantages such as the simple and cheap synthesis of pillararenes, solution-processability, and high thermal and chemical stability. Moreover, the reversible transformations between nonporous guest-free structures and guest-loaded structures make pillararenes highly recyclable. Future work will try to expand the applications of pillararene crystals via host-guest complexation at the solid-solution interface such as liquid-phase separation. Other types of hosts with the potential to encapsulate guests at the solid-solution interface are worth exploring for more unique applications.

## 4. Materials and Methods

### 4.1. Materials

*p*-Diethoxybenzene was purchased from JK Chemicals and used as received. All other chemicals, including tetrahydrofuran (THF) and 1,4-dioxane, were purchased from Sigma-Aldrich and used as received.** EtP5** and** EtP6** were synthesized as described previously [18]. Desolvated crystalline** EtP5** (**EtP5***α*) was recrystallized from acetone and dried under vacuum at 100°C overnight. Desolvated crystalline** EtP6** (**EtP6***β*) was recrystallized from acetone and dried under vacuum at 140°C overnight.

### 4.2. Methods

#### 4.2.1. Solution NMR

Solution ^1^H NMR spectra were recorded at 400.13 MHz using a Bruker Avance 400 NMR spectrometer.

#### 4.2.2. Thermogravimetric Analysis

TGA analysis was carried out using a Q5000IR analyzer (TA instruments) with an automated vertical overhead thermobalance. The samples were heated at the rate of 10°C/min using N_2_ as the protective gas.

#### 4.2.3. Powder X-Ray Diffraction

PXRD data before and after vapor sorption were collected in a Rigaku Ultimate-IV X-ray diffractometer operating at 40 kV/30 mA using the Cu K*α* line (*λ* = 1.5418 Å). Data were measured over the range of 5−40° in 5°/min steps over 7 min.

#### 4.2.4. Single Crystal Growth

Single crystals of dioxane-loaded** EtP6** were grown by a slow evaporation method: 5 mg of dry** EtP6 **powder was put in a small vial where 2 mL of 1,4-dioxane was added. The resultant transparent solution was allowed to evaporate slowly to give nice colorless crystals in 2 to 4 days.

#### 4.2.5. Single Crystal X-Ray Diffraction

Single crystal X-ray data sets were measured on a Rigaku MicroMax-007 HF rotating anode diffractometer (Mo-K*α* radiation,* λ* = 0.71073 Å, Kappa 4-circle goniometer, Rigaku Saturn724+ detector). Unless stated, solvated single crystals, isolated from the crystallization solvent, were immersed in a protective oil, mounted on a MiTeGen loop, and flash-cooled under a dry nitrogen gas flow. Empirical absorption corrections, using the multiscan method, were performed with the program SADABS [36]. Structures were solved with SHELXD [37] or SHELXT [38] or by direct methods using SHELXS [39], refined by full-matrix least squares on |*F*|^2^ by SHELXL [40], and interfaced through the programme OLEX2 [41]. Unless stated, all non-H-atoms were refined anisotropically, and all H-atoms were fixed in geometrically estimated positions and refined using the riding model. Supplementary CIFs, which include structure factors, are available free of charge from the Cambridge Crystallographic Data Centre (CCDC) via www.ccdc.cam.ac.uk/data_request/cif.

#### 4.2.6. Gas Sorption Measurement

Low-pressure gas adsorption measurements were performed on a Micrometritics Accelerated Surface Area and Porosimetry System (ASAP) 2020 surface area analyzer. Samples were degassed under dynamic vacuum for 12 h at 60°C prior to each measurement. N_2_ isotherms were measured using a liquid nitrogen bath (77 K).

## Data Availability

All data needed to evaluate the conclusions in the paper are present in the paper and the Supplementary Materials. Additional data related to this paper may be requested from the authors.

## Supplementary Materials

Supplementary Materials Table S1: experimental single crystal X-ray data for** EtP6 **structure. Figure S1: ball-stick plots from single crystal structures: 4(dioxane)@**E****t****P**6 shown into the cavity (left) and in plane of the aromatic core (right). H-atoms and solvent molecules are omitted for clarity. Not shown on common scale. Figure S2. ^1^H NMR spectrum (400 MHz, CDCl_3_, 293 K) of **E****t****P**5*α*. There is a peak related to protons on H_2_O due to the presence of H_2_O in CDCl_3_. Figure S3: ^1^H NMR spectrum (400 MHz, CDCl_3_, 293 K) of **E****t****P**6*β*. There is a peak related to protons on H_2_O due to the presence of H_2_O in CDCl_3_. Figure S4: thermogravimetric analysis of desolvated** EtP5**. Figure S5: thermogravimetric analysis of desolvated** EtP6**. Figure S6: single crystal structure of guest-free** EtP5**, which is defined as **E****t****P**5*α*  [30]. Figure S7: powder X-ray diffraction pattern: (I) simulated from single crystal structure of guest-free** EtP5**; (II) activated** EtP5** crystals (**E****t****P**5*α*). Figure S8: single crystal structure of thermally stable guest-free** EtP6**, which is defined as **E****t****P**6*β*  [30]. Figure S9: powder X-ray diffraction pattern: (I) activated** EtP6** crystals (**E****t****P**6*β*); (II) simulated from single crystal structure of guest-free** EtP6**. Figure S10. N_2_ adsorption isotherm of **E****t****P**5*α*. Adsorption, closed symbols; desorption, open symbols. Figure S11. N_2_ adsorption isotherm of **E****t****P**6*β*. Adsorption, closed symbols; desorption, open symbols. Figure S12: ^1^H NMR spectrum (400 MHz, D_2_O, 293 K) of a 0.500 mg mL^−1^ D_2_O solution of dioxane after treatment with 1.00 mg of **E****t****P**6*β* for 24 h. Figure S13: ^1^H NMR spectrum (400 MHz, D_2_O, 293 K) of the 0.500 mg mL^−1^ D_2_O solution of dioxane discussed in Figure 12 after further treatment with another 5.00 mg of **E****t****P**6*β* for 24 h. Figure S14: time-dependent partial ^1^H NMR spectra (400 MHz, D_2_O, 293 K) of the dioxane-D_2_O solution upon addition of **E****t****P**5*α*. Figure S15: time-dependent dioxane concentration change in D_2_O upon addition of **E****t****P**5*α*. Figure S16: ^1^H NMR spectrum (400 MHz, CDCl_3_, 293 K) of **E****t****P**5*α* after adsorption of dioxane from water. Figure S17: thermogravimetric analysis of **E****t****P**5*α* after adsorption of dioxane from water. Figure S18: powder X-ray diffraction patterns of** EtP5**: (I) **E****t****P**5*α*; (II) **E****t****P**5*α* after filtration from the 0.500 mg mL^−1^ dioxane-D_2_O solution. Figure S19: ^1^H NMR spectra (400 MHz, CDCl_3_, 293 K): (a) **E****t****P**6*β*; (b) **E****t****P**6*β* after adsorption of dioxane from water; (c) dioxane. Figure S20: ^1^H NMR spectrum (400 MHz, CDCl_3_, 293 K) of **E****t****P**6*β* after adsorption of dioxane from water. The peak area integral can be calculated as two dioxane molecules per** EtP6** molecule. Figure S21: thermogravimetric analysis of **E****t****P**6*β* after adsorption of dioxane from water. The weight loss below 160°C can be calculated as two dioxane molecules per** EtP6** molecule. Figure S22: powder X-ray diffraction patterns of** EtP6**: (I) simulated from single crystal structure of 4(dioxane)@**E****t****P**6; (II) **E****t****P**6*β* filtered from a 1.00 mg mL^−1^ dioxane-D_2_O solution; (III) simulated from single crystal structure of 2(**C****H**)@**E****t****P**6  [25]. Figure S23: time-dependent partial ^1^H NMR spectra (400 MHz, D_2_O, 293 K) of the THF-D_2_O solution upon addition of **E****t****P**5*α*. Figure S24: ^1^H NMR spectrum (400 MHz, D_2_O, 293 K) of a 0.500 mg mL^−1^ D_2_O solution of THF after treatment with 1.00 mg of **E****t****P**5*α* for 24 h. Figure S25: ^1^H NMR spectrum (400 MHz, D_2_O, 293 K) of the 0.500 mg mL^−1^ D_2_O solution of THF discussed in Figure S24 after further treatment with another 1.00 mg of **E****t****P**5*α* for 24 h. Figure S26: time-dependent partial ^1^H NMR spectra (400 MHz, D_2_O, 293 K) of the THF-D_2_O solution upon addition of **E****t****P**6*β*. Figure S27: ^1^H NMR spectrum (400 MHz, 293 K) of a 0.500 mg mL^−1^ D_2_O solution of THF after treatment with 1.00 mg of **E****t****P**6*β* for 24 h. Figure S28: ^1^H NMR spectrum (400 MHz, D_2_O, 293 K) of the 0.500 mg mL^−1^ D_2_O solution of THF discussed in Figure S27 after further treatment with another 1.00 mg of **E****t****P**6*β* for 24 h. Figure S29: ^1^H NMR spectrum (400 MHz, CDCl_3_, 293 K) of **E****t****P**5*α* after adsorption of THF from water. Figure S30: partial ^1^H NMR spectra (400 MHz, CDCl_3_, 293 K): (a) **E****t****P**5*α*; (b) **E****t****P**5*α* after adsorption of THF from water; (c) THF. Figure S31: thermogravimetric analysis of **E****t****P**5*α* after adsorption of THF from water. The weight loss below 100°C can be calculated as two THF molecules per** EtP5** molecule. Figure S32: ^1^H NMR spectrum (400 MHz, CDCl_3_, 293 K) of **E****t****P**6*β* after adsorption of THF from water. Figure S33: partial ^1^H NMR spectra (400 MHz, CDCl_3_, 293 K): (a) **E****t****P**6*β*; (b) **E****t****P**6*β* after adsorption of THF from water; (c) THF. Figure S34: thermogravimetric analysis of **E****t****P**6*β* after adsorption of dioxane from water. The weight loss below 100°C can be calculated as one THF molecule per** EtP6** molecule. Figure S35: time-dependent ^1^H NMR spectra (400 MHz, D_2_O, 293 K) of a D_2_O solution (0.600 mL) of 0.500 mg mL^−1^ dioxane and 0.500 mg mL^−1^ THF after treatment with 5.00 mg of **E****t****P**6*β* for 24 h. Figure S36: ^1^H NMR spectrum (400 MHz, CDCl_3_, 293 K) of desolvated** EtP5** upon removal of THF. Figure S37: thermogravimetric analysis of desolvated** EtP5** upon removal of THF. Figure S38: powder X-ray diffraction patterns of** EtP5**: (I) **E****t****P**5*α*; (II) desolvated 2(THF)@**E****t****P**5  [28]. This implies that upon removal of THF, 2(THF)@**E****t****P**5 transforms back to **E****t****P**5*α*. Figure S39: ^1^H NMR spectrum (400 MHz, CDCl_3_, 293 K) of desolvated** EtP6** upon removal of dioxane. Figure S40: ^1^H NMR spectrum (400 MHz, CDCl_3_, 293 K) of desolvated** EtP6** upon removal of THF. Figure S41: thermogravimetric analysis of desolvated** EtP6** upon removal of dioxane. Figure S42: thermogravimetric analysis of desolvated** EtP6** upon removal of THF. Figure S43: powder X-ray diffraction patterns of** EtP6**: (I) desolvated THF@**E****t****P**6; (II) desolvated 2(dioxane)@**E****t****P**6; (III) **E****t****P**6*β*. This implies that upon removal of THF or dioxane, THF@**E****t****P**6 and 2(dioxane)@**E****t****P**6 transform back to **E****t****P**6*β* [25, 28, 30].
