# Supplementary material for: Cyclic Ether Contaminant Removal from Water Using Nonporous Adaptive Pillararene Crystals via Host-Guest Complexation at the Solid-Solution Interface
Source: Research (Wash D C). 2019 May 12;2019:5406365. doi: 10.34133/2019/5406365 (PMC6750096; doi:10.34133/2019/5406365)
Supplement: Supplementary Materials — Table S1: experimental single crystal X-ray data for EtP6 structure. Figure S1: ball-stick plots from single crystal structures: 4(dioxane)@EtP6 shown into the cavity (left) and in plane of the aromatic core (right). H-atoms and solvent molecules are omitted for clarity. Not shown on common scale. Figure S2. 1H NMR spectrum (400 MHz, CDCl3, 293 K) of EtP5α. There is a peak related to protons on H2O due to the presence of H2O in CDCl3. Figure S3: 1H NMR spectrum (400 MHz, CDCl3, 293 K) of EtP6β. There is a peak related to protons on H2O due to the presence of H2O in CDCl3. Figure S4: thermogravimetric analysis of desolvated EtP5. Figure S5: thermogravimetric analysis of desolvated EtP6. Figure S6: single crystal structure of guest-free EtP5, which is defined as EtP5α [30]. Figure S7: powder X-ray diffraction pattern: (I) simulated from single crystal structure of guest-free EtP5; (II) activated EtP5 crystals (EtP5α). Figure S8: single crystal structure of thermally stable guest-free EtP6, which is defined as EtP6β [30]. Figure S9: powder X-ray diffraction pattern: (I) activated EtP6 crystals (EtP6β); (II) simulated from single crystal structure of guest-free EtP6. Figure S10. N2 adsorption isotherm of EtP5α. Adsorption, closed symbols; desorption, open symbols. Figure S11. N2 adsorption isotherm of EtP6β. Adsorption, closed symbols; desorption, open symbols. Figure S12: 1H NMR spectrum (400 MHz, D2O, 293 K) of a 0.500 mg mL−1 D2O solution of dioxane after treatment with 1.00 mg of EtP6β for 24 h. Figure S13: 1H NMR spectrum (400 MHz, D2O, 293 K) of the 0.500 mg mL−1 D2O solution of dioxane discussed in Figure 12 after further treatment with another 5.00 mg of EtP6β for 24 h. Figure S14: time-dependent partial 1H NMR spectra (400 MHz, D2O, 293 K) of the dioxane-D2O solution upon addition of EtP5α. Figure S15: time-dependent dioxane concentration change in D2O upon addition of EtP5α. Figure S16: 1H NMR spectrum (400 MHz, CDCl3, 293 K) of EtP5α after adsorption of dioxa [file 5406365.f1.zip › 5406365_SupplDesc.docx]

**Table S1**. Experimental single crystal X-ray data for **EtP6** structure. **Figure S1**. Ball-stick plots from single crystal structures: 4(dioxane)@**EtP6** shown into the cavity (left) and in plane of the aromatic core (right). H-atoms and solvent molecules are omitted for clarity. Not shown on common scale. **Figure S2**. ^1^H NMR spectrum (400 MHz, CDCl_3_, 293 K) of **EtP5***α*. There is a peak related to protons on H_2_O due to the presence of H_2_O in CDCl_3_. **Figure S3**. ^1^H NMR spectrum (400 MHz, CDCl_3_, 293 K) of **EtP6***β*. There is a peak related to protons on H_2_O due to the presence of H_2_O in CDCl_3_. **Figure S4**. Thermogravimetric analysis of desolvated **EtP5**. **Figure S5**. Thermogravimetric analysis of desolvated **EtP6**. **Figure S6**. Single crystal structure of guest-free **EtP5**, which is defined as **EtP5***α* [31]. **Figure S7**. Powder X-ray diffraction pattern: (I) simulated from single crystal structure of guest-free **EtP5**; (II) activated **EtP5** crystals (**EtP5***α*). **Figure S8** Single crystal structure of thermally stable guest-free **EtP6**, which is defined as **EtP6***β* [31]. **Figure S9**. Powder X-ray diffraction pattern: (I) activated **EtP6** crystals (**EtP6***β*); (II) simulated from single crystal structure of guest-free **EtP6**. **Figure S10**. N_2_ adsorption isotherm of **EtP5***α*. Adsorption, closed symbols; desorption, open symbols. **Figure S11**. N_2_ adsorption isotherm of **EtP6***β*. Adsorption, closed symbols; desorption, open symbols. **Figure S12**. ^1^H NMR spectrum (400 MHz, D_2_O, 293 K) of a 0.500 mg mL^−1^ D_2_O solution of dioxane after treatment with 1.00 mg of **EtP6***β* for 24 h. **Figure S13**. ^1^H NMR spectrum (400 MHz, D_2_O, 293 K) of the 0.500 mg mL^−1^ D_2_O solution of dioxane discussed in Figure 12 after further treatment with another 5.00 mg of **EtP6***β* for 24 h. **Figure S14**. Time-dependent partial ^1^H NMR spectra (400 MHz, D_2_O, 293 K) of the dioxane-D_2_O solution upon addition of **EtP5***α*. **Figure S15**. Time-dependent dioxane concentration change in D_2_O upon addition of **EtP5***α*. **Figure S16**. ^1^H NMR spectrum (400 MHz, CDCl_3_, 293 K) of **EtP5***α* after adsorption of dioxane from water. **Figure S17**. Thermogravimetric analysis of **EtP5***α* after adsorption of dioxane from water. **Figure S18**. Powder X-ray diffraction patterns of **EtP5**: (I) **EtP5***α*; (II) **EtP5***α* after filtration from the 0.500 mg mL^−1^ dioxane-D_2_O solution. **Figure S19**. ^1^H NMR spectra (400 MHz, CDCl_3_, 293 K): (a) **EtP6***β*; (b) **EtP6***β* after adsorption of dioxane from water; (c) dioxane. **Figure S20**. ^1^H NMR spectrum (400 MHz, CDCl_3_, 293 K) of **EtP6***β* after adsorption of dioxane from water. The peak area integral can be calculated as two dioxane molecules per **EtP6** molecule. **Figure S21** Thermogravimetric analysis of **EtP6***β* after adsorption of dioxane from water. The weight loss below 160 °C can be calculated as two dioxane molecules per **EtP6** molecule. **Figure S22**. Powder X-ray diffraction patterns of **EtP6**: (I) simulated from single crystal structure of 4(dioxane)@**EtP6**; (II) **EtP6***β* filtered from a 1.00 mg mL^−1^ dioxane-D_2_O solution; (III) simulated from single crystal structure of 2(**CH**)@**EtP6** [26]. **Figure S23**. Time-dependent partial ^1^H NMR spectra (400 MHz, D_2_O, 293 K) of the THF-D_2_O solution upon addition of **EtP5***α*. **Figure S24**. ^1^H NMR spectrum (400 MHz, D_2_O, 293 K) of a 0.500 mg mL^−1^ D_2_O solution of THF after treatment with 1.00 mg of **EtP5***α* for 24 h. **Figure S25**. ^1^H NMR spectrum (400 MHz, D_2_O, 293 K) of the 0.500 mg mL^−1^ D_2_O solution of THF discussed in Figure S24 after further treatment with another 1.00 mg of **EtP5***α* for 24 h. **Figure S26**. Time-dependent partial ^1^H NMR spectra (400 MHz, D_2_O, 293 K) of the THF-D_2_O solution upon addition of **EtP6***β*. **Figure S27**. ^1^H NMR spectrum (400 MHz, 293 K) of a 0.500 mg mL^−1^ D_2_O solution of THF after treatment with 1.00 mg of **EtP6***β* for 24 h. **Figure S28**. ^1^H NMR spectrum (400 MHz, D_2_O, 293 K) of the 0.500 mg mL^−1^ D_2_O solution of THF discussed in Figure S27 after further treatment with another 1.00 mg of **EtP6***β* for 24 h. **Figure S29**. ^1^H NMR spectrum (400 MHz, CDCl_3_, 293 K) of **EtP5***α* after adsorption of THF from water. **Figure S30**. Partial ^1^H NMR spectra (400 MHz, CDCl_3_, 293 K): (a) **EtP5***α*; (b) **EtP5***α* after adsorption of THF from water; (c) THF. **Figure S31**. Thermogravimetric analysis of **EtP5***α* after adsorption of THF from water. The weight loss below 100 °C can be calculated as two THF molecules per **EtP5** molecule. **Figure S32**. ^1^H NMR spectrum (400 MHz, CDCl_3_, 293 K) of **EtP6***β* after adsorption of THF from water. **Figure S33**. Partial ^1^H NMR spectra (400 MHz, CDCl_3_, 293 K): (a) **EtP6***β*; (b) **EtP6***β* after adsorption of THF from water; (c) THF. **Figure S34**. Thermogravimetric analysis of **EtP6***β* after adsorption of dioxane from water. The weight loss below 100 °C can be calculated as one THF molecule per **EtP6** molecule. **Figure S35**. Time-dependent ^1^H NMR spectra (400 MHz, D_2_O, 293 K) of a D_2_O solution (0.600 mL) of 0.500 mg mL^−1^ dioxane and 0.500 mg mL^−1^ THF after treatment with 5.00 mg of **EtP6***β* for 24 h. **Figure S36**. ^1^H NMR spectrum (400 MHz, CDCl_3_, 293 K) of desolvated **EtP5** upon removal of THF. **Figure S37**. Thermogravimetric analysis of desolvated **EtP5** upon removal of THF. **Figure S38**. Powder X-ray diffraction patterns of **EtP5**: (I) **EtP5***α*; (II) desolvated 2(THF)@**EtP5** [29]. This implies that upon removal of THF, 2(THF)@**EtP5** transforms back to **EtP5***α*. **Figure S39**. ^1^H NMR spectrum (400 MHz, CDCl_3_, 293 K) of desolvated **EtP6** upon removal of dioxane. **Figure S40**. ^1^H NMR spectrum (400 MHz, CDCl_3_, 293 K) of desolvated **EtP6** upon removal of THF. **Figure S41**. Thermogravimetric analysis of desolvated **EtP6** upon removal of dioxane. **Figure S42**. Thermogravimetric analysis of desolvated **EtP6** upon removal of THF. **Figure S43**. Powder X-ray diffraction patterns of **EtP6**: (I) desolvated THF@**EtP6**; (II) desolvated 2(dioxane)@**EtP6**; (III) **EtP6***β*. This implies that upon removal of THF or dioxane, THF@**EtP6** and 2(dioxane)@**EtP6** transform back to **EtP6***β*. References [26], [29], [31] (Supplementary Materials)
