# Supplementary material for: Cyclic Ether Contaminant Removal from Water Using Nonporous Adaptive Pillararene Crystals via Host-Guest Complexation at the Solid-Solution Interface
Source: Research (Wash D C). 2019 May 12;2019:5406365. doi: 10.34133/2019/5406365 (PMC6750096; doi:10.34133/2019/5406365)
Supplement: Supplementary Materials — Table S1: experimental single crystal X-ray data for EtP6 structure. Figure S1: ball-stick plots from single crystal structures: 4(dioxane)@EtP6 shown into the cavity (left) and in plane of the aromatic core (right). H-atoms and solvent molecules are omitted for clarity. Not shown on common scale. Figure S2. 1H NMR spectrum (400 MHz, CDCl3, 293 K) of EtP5α. There is a peak related to protons on H2O due to the presence of H2O in CDCl3. Figure S3: 1H NMR spectrum (400 MHz, CDCl3, 293 K) of EtP6β. There is a peak related to protons on H2O due to the presence of H2O in CDCl3. Figure S4: thermogravimetric analysis of desolvated EtP5. Figure S5: thermogravimetric analysis of desolvated EtP6. Figure S6: single crystal structure of guest-free EtP5, which is defined as EtP5α [30]. Figure S7: powder X-ray diffraction pattern: (I) simulated from single crystal structure of guest-free EtP5; (II) activated EtP5 crystals (EtP5α). Figure S8: single crystal structure of thermally stable guest-free EtP6, which is defined as EtP6β [30]. Figure S9: powder X-ray diffraction pattern: (I) activated EtP6 crystals (EtP6β); (II) simulated from single crystal structure of guest-free EtP6. Figure S10. N2 adsorption isotherm of EtP5α. Adsorption, closed symbols; desorption, open symbols. Figure S11. N2 adsorption isotherm of EtP6β. Adsorption, closed symbols; desorption, open symbols. Figure S12: 1H NMR spectrum (400 MHz, D2O, 293 K) of a 0.500 mg mL−1 D2O solution of dioxane after treatment with 1.00 mg of EtP6β for 24 h. Figure S13: 1H NMR spectrum (400 MHz, D2O, 293 K) of the 0.500 mg mL−1 D2O solution of dioxane discussed in Figure 12 after further treatment with another 5.00 mg of EtP6β for 24 h. Figure S14: time-dependent partial 1H NMR spectra (400 MHz, D2O, 293 K) of the dioxane-D2O solution upon addition of EtP5α. Figure S15: time-dependent dioxane concentration change in D2O upon addition of EtP5α. Figure S16: 1H NMR spectrum (400 MHz, CDCl3, 293 K) of EtP5α after adsorption of dioxa [file 5406365.f1.zip › Supplementary Materials.doc]

Supplementary Materials

**1. Crystallography Data**

**Table S1**. Experimental single crystal X-ray data for **EtP6** structure.

| Formula | **EtP6**∙4(1,4-dioxane) |
| --- | --- |
| Crystallization Solvent | 1,4-dioxane |
| Collection Temperature | 173 K |
| Formula | C_90_H_132_O_24_ |
| *Mr* | 1597.95 |
| Crystal Size [mm] | 0.362 x 0.302 x 0.128 |
| Crystal System | Triclinic |
| Space Group | P |
| *a* [Å] | 11.161(3) |
| *b* [Å] | 12.5586(14) |
| *c* [Å] | 16.376(4) |
| *α* [°] | 100.670(2) |
| *β* [°] | 97.306(18) |
| *γ* [°] | 102.668(14) |
| *V* [Å^3^] | 2166.8 (8) |
| *Z* | 1 |
| D*_calcd_* [g cm^-3^] | 1.225 |
| *μ*[mm^-1^] | 0.714 |
| F(000) | 864 |
| 2*θ* range [°] | 5.58 – 133.774 |
| Reflections collected | 49729 |
| Independent reflections, *R_int_* | 7556, 0.0226 |
| Obs. Data [*I* > *2σ*(*I*)] | 6153 |
| Data /restraints /parameters | 7556 / 0 / 520 |
| Final *R*_1_ values (*I* > 2*σ*(*I*)) | 0.0480 |
| Final *R*_1_ values (all data) | 0.0606 |
| Final *wR*(*F*_2_) values (all data) | 0.1345 |
| ­Goodness-of-fit on *F*^2^ | 1.046 |
| Largest difference peak and hole [e.A^-3^] | 0.603 / -0.38 |
| CCDC | 1584743 |

**Figure S1**. Ball-stick plots from single crystal structures: 4(dioxane)@**EtP6** shown into the cavity (left) and in plane of the aromatic core (right). H-atoms and dioxane molecules are omitted for clarity. Not shown on common scale.

**2. Characterization of Desolvated EtP5 Crystals (EtP5*α*) and EtP6 crystals (EtP6*β*)**

**Figure S2**. ^1^H NMR spectrum (400 MHz, CDCl_3_, 293 K) of **EtP5***α*. There is a peak related to protons on H_2_O due to the presence of H_2_O in CDCl_3_.

**Figure S3**. ^1^H NMR spectrum (400 MHz, CDCl_3_, 293 K) of **EtP6***β*. There is a peak related to protons on H_2_O due to the presence of H_2_O in CDCl_3_.

**Figure S4**. Thermogravimetric analysis of desolvated **EtP5**.

**Figure S5**. Thermogravimetric analysis of desolvated **EtP6**.

**Figure S6**. Single crystal structure of guest-free **EtP5**, which is defined as **EtP5***α* [31].

**Figure S7**. Powder X-ray diffraction pattern: (I) simulated from single crystal structure of guest-free **EtP5**; (II) activated **EtP5** crystals (**EtP5***α*).

**Figure S8**. Single crystal structure of thermally stable guest-free **EtP6**, which is defined as **EtP6***β* [31].

**Figure S9**. Powder X-ray diffraction pattern: (I) activated **EtP6** crystals (**EtP6***β*); (II) simulated from single crystal structure of guest-free **EtP6**.

**Figure S10**. N_2_ adsorption isotherm of **EtP5***α*. Adsorption, closed symbols; desorption, open symbols.

**Figure S11**. N_2_ adsorption isotherm of **EtP6***β*. Adsorption, closed symbols; desorption, open symbols.

**3. Dioxane Aqueous Solution Adsorption Measurements**

Dioxane was dissolved in D_2_O (0.600 mL) with a concentration of 0.500 mg mL^−1^ (5.7 × 10^−3^ mmol mL^−1^), where 1.00 mg of water-insoluble **EtP5***α* and **EtP6***β* crystals were added, respectively. The content of dioxane in D_2_O was recorded by time-dependent ^1^H NMR.

**Figure S12**. ^1^H NMR spectrum (400 MHz, D_2_O, 293 K) of a 0.500 mg mL^−1^ D_2_O solution of dioxane after treatment with 1.00 mg of **EtP6***β* for 24 h.

**Figure S13**. ^1^H NMR spectrum (400 MHz, D_2_O, 293 K) of the 0.500 mg mL^−1^ D_2_O solution of dioxane discussed in Figure S12 after further treatment with another 5.00 mg of **EtP6***β* for 24 h.

**3.1. Time-dependent Partial ^1^H NMR Spectra of the Dioxane-D_2_O Solution upon Addition of EtP5*α***

**Figure S14**. Time-dependent partial ^1^H NMR spectra (400 MHz, D_2_O, 293 K) of the dioxane-D_2_O solution upon addition of **EtP5***α*.

**Figure S15**. Time-dependent dioxane concentration change in D_2_O upon addition of **EtP5***α*.

**3.2 Characterization of EtP5*α* after Adsorption of Dioxane from Water**

^1^H NMR experiments were performed by dissolving **EtP5***α* crystals filtered from the dioxane aqueous solution in CDCl_3_. TGA and PXRD experiments were performed using **EtP5***α* crystals filtered from the dioxane aqueous solution.

**Figure S16**. ^1^H NMR spectrum (400 MHz, CDCl_3_, 293 K) of **EtP5***α* after adsorption of dioxane from water.

**Figure S17**. Thermogravimetric analysis of **EtP5***α* after adsorption of dioxane from water.

**Figure S18**. Powder X-ray diffraction patterns of **EtP5**: (I) **EtP5***α*; (II) **EtP5***α* after filtration from the 0.500 mg mL^−1^ dioxane-D_2_O solution.

**3.3. Characterization of EtP6*β* after Adsorption of Dioxane from Water**

^1^H NMR experiments were performed by dissolving **EtP6***β* crystals filtered from the dioxane aqueous solution in CDCl_3_. TGA and PXRD experiments using **EtP6***β* crystals after filtered from the dioxane aqueous solution.

**Figure S19**. ^1^H NMR spectra (400 MHz, CDCl_3_, 293 K): (a) **EtP6***β*; (b) **EtP6***β* after adsorption of dioxane from water; (c) dioxane.

**Figure S20**. ^1^H NMR spectrum (400 MHz, CDCl_3_, 293 K) of **EtP6***β* after adsorption of dioxane from water. The peak area integral can be calculated as two dioxane molecules per **EtP6** molecule.

**Figure S21**. Thermogravimetric analysis of **EtP6***β* after adsorption of dioxane from water. The weight loss below 160 °C can be calculated as two dioxane molecules per **EtP6** molecule.

**Figure S22**. Powder X-ray diffraction patterns of **EtP6**: (I) simulated from single crystal structure of 4(dioxane)@**EtP6**; (II) **EtP6***β* filtered from a 1.00 mg mL^−1^ dioxane-D_2_O solution; (III) simulated from single crystal structure of 2(**CH**)@**EtP6** [26].

**4. Tetrahydrofuran (THF) Aqueous Solution Adsorption Measurements**

THF was firstly dissolved in D_2_O (0.600 mL) with a concentration of 0.500 mg mL^−1^ (6.90 × 10^−3^ mmol mL^−1^), where 1.00 mg of water-insoluble **EtP5***α* or **EtP6***β* crystals were added, respectively. The content of THF in D_2_O was recorded by time-dependent ^1^H NMR.

**4.1. Time-dependent Partial ^1^H NMR Spectra of the THF-D_2_O Solution upon Addition of EtP5*α***

**Figure S23**. Time-dependent partial ^1^H NMR spectra (400 MHz, D_2_O, 293 K) of the THF-D_2_O solution upon addition of **EtP5***α*.

**Figure S24**. ^1^H NMR spectrum (400 MHz, D_2_O, 293 K) of a 0.500 mg mL^−1^ D_2_O solution of THF after treatment with 1.00 mg of **EtP5***α* for 24 h.

**Figure S25**. ^1^H NMR spectrum (400 MHz, D_2_O, 293 K) of the 0.500 mg mL^−1^ D_2_O solution of THF discussed in Figure S24 after further treatment with another 1.00 mg of **EtP5***α* for 24 h.

**4.2. Time-dependent Partial ^1^H NMR Spectra of the THF-D_2_O Solution upon Addition of EtP6*β***

**Figure S26**. Time-dependent partial ^1^H NMR spectra (400 MHz, D_2_O, 293 K) of the THF-D_2_O solution upon addition of **EtP6***β*.

**Figure S27**. ^1^H NMR spectrum (400 MHz, 293 K) of a 0.500 mg mL^−1^ D_2_O solution of THF after treatment with 1.00 mg of **EtP6***β* for 24 h.

**Figure S28**. ^1^H NMR spectrum (400 MHz, D_2_O, 293 K) of the 0.500 mg mL^−1^ D_2_O solution of THF discussed in Figure 27 after further treatment with another 1.00 mg of **EtP6***β* for 24 h.

**4.3 Characterization of EtP5*α* after Adsorption of THF from Water**

^1^H NMR experiments were performed by dissolving **EtP5***α* crystals which were filtered after adsorption of THF from water in CDCl_3_. TGA and PXRD experiments were performed using **EtP5***α* crystals after adsorption of THF from water.

**Figure S29**. ^1^H NMR spectrum (400 MHz, CDCl_3_, 293 K) of **EtP5***α* after adsorption of THF from water.

**Figure S30**. Partial ^1^H NMR spectra (400 MHz, CDCl_3_, 293 K): (a) **EtP5***α*; (b) **EtP5***α* after adsorption of THF from water; (c) THF.

**Figure S31**. Thermogravimetric analysis of **EtP5***α* after adsorption of THF from water. The weight loss below 100 °C can be calculated as two THF molecules per **EtP5** molecule.

**4.4 Characterization of EtP6*β* after Adsorption of THF from Water**

^1^H NMR experiments were performed by dissolving **EtP6***β* crystals which were filtered after adsorption of THF from water in CDCl_3_. TGA and PXRD experiments were performed using **EtP6***β* crystals after adsorption of THF from water.

**Figure S32**. ^1^H NMR spectrum (400 MHz, CDCl_3_, 293 K) of **EtP6***β* after adsorption of THF from water.

**Figure S33**. Partial ^1^H NMR spectra (400 MHz, CDCl_3_, 293 K): (a) **EtP6***β*; (b) **EtP6***β* after adsorption of THF from water; (c) THF.

**Figure S34**. Thermogravimetric analysis of **EtP6***β* after adsorption of dioxane from water. The weight loss below 100 °C can be calculated as one THF molecule per **EtP6** molecule.

**5. Selective Removal of Dioxane in the Presence of THF**

**Figure S35**. Time-dependent ^1^H NMR spectra (400 MHz, D_2_O, 293 K) of a D_2_O solution (0.600 mL) of 0.500 mg mL^−1^ dioxane and 0.500 mg mL^−1^ THF after treatment with 5.00 mg of **EtP6***β* for 24 h.

**6. Recyclability of EtP5*α* and EtP6*β* Crystals**

An open 5.00 mL vial containing 0.100 g of 2(THF)@**EtP5** was desolvated under vacuum at 100 °C overnight. An open 5.00 mL vial containing 0.100 g of THF@**EtP6** and 2(dioxane)@**EtP6** was desolvated under vacuum at 120 °C overnight. The resultant crystals were characterized by ^1^H NMR, TGA and PXRD.

**Figure S36**. ^1^H NMR spectrum (400 MHz, CDCl_3_, 293 K) of desolvated **EtP5** upon removal of THF.

**Figure S37**. Thermogravimetric analysis of desolvated **EtP5** upon removal of THF.

**Figure S38**. Powder X-ray diffraction patterns of **EtP5**: (I) **EtP5***α*; (II) desolvated 2(THF)@**EtP5** [29]. This implies that upon removal of THF, 2(THF)@**EtP5** transforms back to **EtP5***α*.

**Figure S39**. ^1^H NMR spectrum (400 MHz, CDCl_3_, 293 K) of desolvated **EtP6** upon removal of dioxane.

**Figure S40**. ^1^H NMR spectrum (400 MHz, CDCl_3_, 293 K) of desolvated **EtP6** upon removal of THF.

**Figure S41**. Thermogravimetric analysis of desolvated **EtP6** upon removal of dioxane.

**Figure S42**. Thermogravimetric analysis of desolvated **EtP6** upon removal of THF.

**Figure S43**. Powder X-ray diffraction patterns of **EtP6**: (I) desolvated THF@**EtP6**; (II) desolvated 2(dioxane)@**EtP6**; (III) **EtP6***β*. This implies that upon removal of THF or dioxane, THF@**EtP6** and 2(dioxane)@**EtP6** transform back to **EtP6***β*.
